# The Evolutionary Origins of the Southern Ocean Philobryid Bivalves: Hidden Biodiversity, Ancient Persistence

**DOI:** 10.1371/journal.pone.0121198

**Published:** 2015-04-08

**Authors:** Jennifer A. Jackson, Katrin Linse, Rowan Whittle, Huw J. Griffiths

**Affiliations:** British Antarctic Survey, High Cross, Madingley Road, Cambridge, CB3 0ET, United Kingdom; University of California, UNITED STATES

## Abstract

Philobryids (Bivalvia: Arcoida) are one of the most speciose marine bivalve families in the Southern Ocean and are common throughout the Southern Hemisphere. Considering this diversity and their brooding reproductive mode (limiting long-distance dispersal), this family may have been present in the Southern Ocean since its inception. However *Philobrya* and *Adacnarca* appear only in the Quaternary fossil record of the Antarctic, suggesting a much more recent incursion. Molecular dating provides an independent means of measuring the time of origin and radiation of this poorly known group. Here we present the first combined molecular and morphological investigation of the Philobryidae in the Southern Ocean. Two nuclear loci (*18S* and *28S*) were amplified from 35 Southern Ocean *Adacnarca* and *Philobrya* specimens, with a combined sequence length of 2,282 base pairs (bp). *Adacnarca* specimens (*A*. *nitens* and *A*. *limopsoides*) were resolved as a strongly supported monophyletic group. Genus *Philobrya* fell into two strongly supported groups (‘*sublaevis*’ and ‘*magellanica/wandelensis*’), paraphyletic with *Adacnarca*. The *A*. *nitens* species complex is identified as at least seven morpho-species through morphological and genetic analysis of taxon clustering. Phylogenetic analyses resolve Philobryidae as a strongly supported monophyletic clade and sister taxon to the Limopsidae, as anticipated by their classification into the superfamily Limopsoidea. Bayesian relaxed clock analyses of divergence times suggest that genus *Adacnarca* radiated in the Southern Ocean from the Early Paleogene, while *P*. *sublaevis* and *P*. *wandelensis* clades radiated in the late Miocene, following the formation of the Antarctic Circumpolar Current.

## Introduction

The Southern Ocean is a unique and isolated marine habitat, with over-deepened continental shelves, oceanography strongly influenced by the circum-Antarctic current and a low-temperature, stenothermal environment. This ocean is also home to a great number of endemic and unusual species which have survived multiple glacial cycles, often in fragmented populations within the Southern Ocean seascape. A great deal of Southern Ocean diversity is still unknown; recent initiatives such as the Census of Antarctic Marine life have helped to increase the rate of description of some of these species [1] but the described Southern Ocean diversity is considered to be greatly underestimated for most fauna [2]. For many taxonomic groups therefore, the crucial first step of identifying species both morphologically and genetically is still being undertaken.

Global marine mollusk diversity is poorly represented in the Antarctic marine realm compared to the non-Antarctic: analyses of latitudinal species diversity show a strong downward cline from the tropics toward the poles [3]. Within Bivalvia, an estimated 136 species are present in the Southern Ocean in comparison with c. 10,000 species worldwide [4]. The Southern Ocean bivalve fauna is dominated by pteriomorphs and heterodonts, but none of these genera are particularly speciose, with a median species diversity of 2.1 (range 1–12) for genera south of the Polar Front (from www.biodiversity.aq) [5]. At the family and genus levels, the poorly known pteriomorph Philobryidae are one of the most successful Southern Ocean bivalve groups, with 14 species identified south of the Polar Front. This family is found across the Southern Hemisphere in a wide but patchy distribution, and is particularly common in the waters off New Zealand, Australia and Antarctica. Philobryids are small in size (<1.5cm), mytiliform and epibyssate, and occur from the intertidal zone to deep waters >1000m. The genus *Philobrya* is the second-most speciose bivalve genus in the Southern Ocean (after *Limopsis*), with nine species found south of the Polar Front [5].

According to the fossil record, the evolutionary origins of this genus are in the Paleogene, with philobryids first appearing in the Eocene, and members of *Philobrya* and *Lissarca* both found in the Miocene [6]. Despite the relative diversity of Southern Ocean philobryids, the family is only known from the Quaternary in the Antarctic fossil record [7], suggesting that radiation of these species into the Southern Ocean may have happened very recently. Philobryids are a viviparous family, and some observations suggest that they brood young to a fairly large size [6], which indicates that they may have limited dispersal ability. They are also epibyssate, able to attach to geological and biological substrates such as rocks, seaweeds, hydrozoans or cidaroid urchins [8–11]. Attachment to more mobile fauna may serve as a mechanism for longer-range dispersal of members of this family.

As benthic brooders with limited dispersal abilities, it is possible that the Southern Hemisphere Philobryidae may have tracked continental drift, with the break up of Gondwana and the isolation of Antarctica. In order to investigate the evolutionary origins and radiation of this poorly known family in the Southern Ocean, we have sequenced two nuclear loci (*18S* and *28S*; 2282bp) from 35 specimens distributed across the Falkland Islands, Weddell Sea, Scotia Sea and Antarctic Peninsula. These nuclear loci were chosen in order to investigate the deep-time inter-genera and inter-family evolutionary relationships of the Philobryidae within the Arcoida. Following morphological identification of these specimens, generalized mixed Yule coalescent and Automatic Barcode Gap Discovery models were used to cluster *28S* genetic lineages into multiple clades representing at least 14 putative species. In order to place the Philobryidae into a broader taxonomic context, we use Bayesian and maximum likelihood phylogenetic approaches to examine for the first time the relationship of this family to other arcoids and limopsids within the bivalve order Arcoida. We then integrate this phylogenetic approach with available fossil data to produce the first time-calibrated measure of inter-species divergence within the Arcoida, and estimate the timeframe over which the Philobryidae radiated into the Southern Ocean and diverged from other arcoids within this poorly known order.

## Materials and Methods

### Sample collection

**Fig 1:**
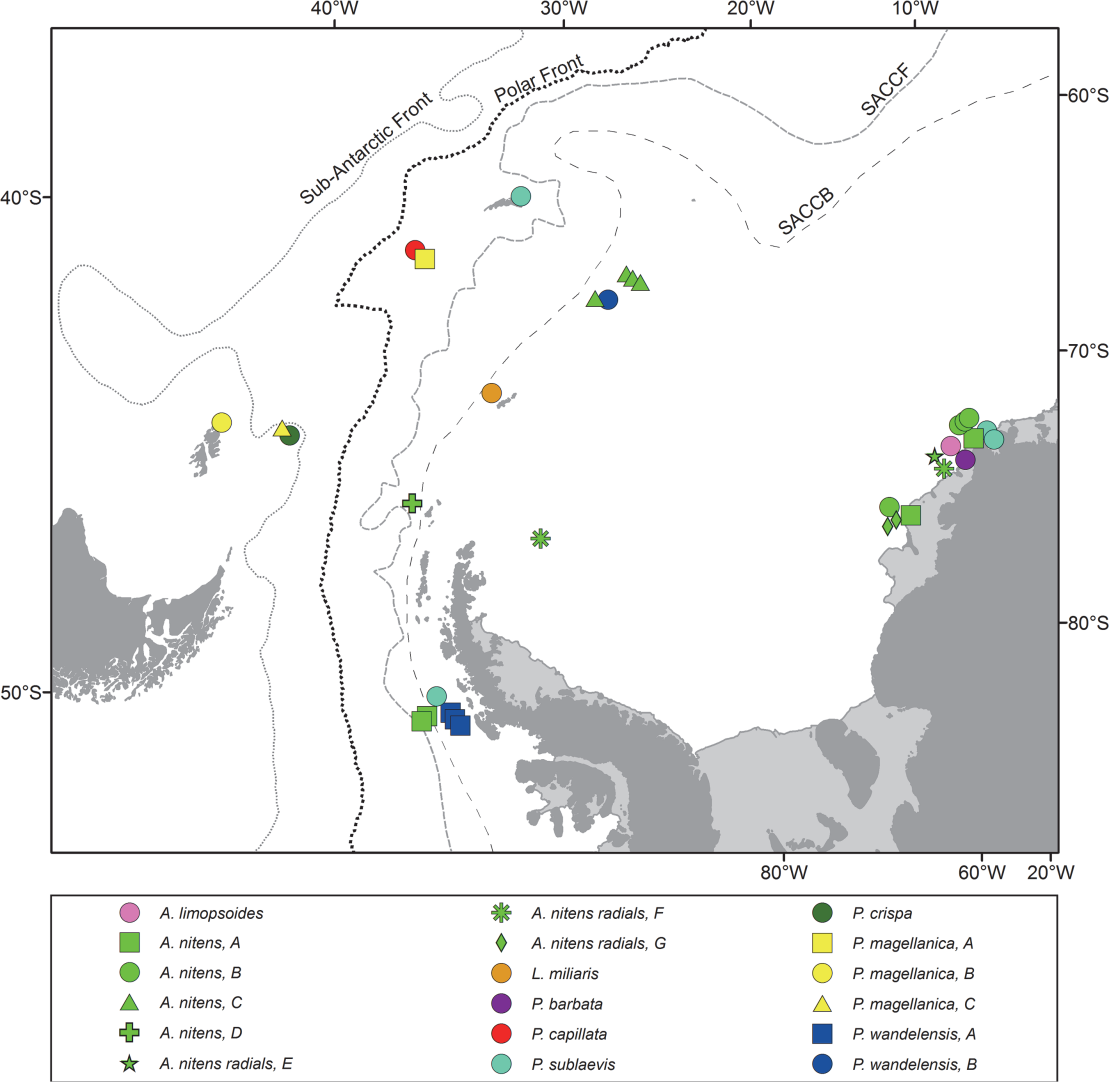
Locations of specimens collected for this study.

Philobryid bivalves were collected during a dive expedition to Rothera Research Station and the Falkland Islands in 2001 and three RV Polarstern expeditions ANDEEP II, LAMPOS and BENDEX to the Scotia and Weddell seas in 2002 and 2003/04 [12–14] (Table 1, Fig. 1). Specimens were fixed in pre-cooled, 96% ethanol immediately subsequent to collection and kept at −20°C until tissue dissection for analysis.

**Table 1:** Antarctic Philobryidae analyzed in this study.

	Specimen Number	GenBank Accession #		Sample collection code	Depth	Latitude/longitude
		*18S*	*28S*			
***Philobrya***						
*P*. *capillata*	02–728	KP340852		PS61–164	317	53°23.80`S 042°42.03`W
*P*. *crispa*	02–498	KP340859	KP340806	PS61–150	288	54°30.22`S 056°08.20`W
*P*. *wandelensis A*	01–41	KP340854	KP340815	Rothera South Cove	0–30m	65°34’09’S 068°07’54W
	01-08-2	KP340856	KP340814	Rothera South Cove	0–30m	65°34’09’S 068°07’54W
	01–55	KP340857	KP340816	Rothera South Cove	0–30m	65°34’09’S 068°07’54W
*P*. *wandelensis B*	02–692		KP340813	PS61–223	376	60°08.16`S 034°55.59`W
*P*. *magellanica A*	02–600	KP340855	KP340812	PS61–164	317	53°23.80`S 042°42.03`W
*P*. *magellanica B*	01-81-1	KP340835		Falkland Islands		51°40.33`S 057°41.17`W
	01-81-2	KP340853	KP340811	Falkland Islands		51°40.33`S 057°41.17`W
*P*. *magellanica C*	02–558	KP340858		PS61–150	288	54°30.22`S 056°08.20`W
*P*. *sublaevis*	02–659	KP340844	KP340809	PS61–182	253	54°27.63`S 035°41.33`W
	01–43	KP340846	KP340808	Rothera South Cove	0–30m	65°34’09’S 068°07’54W
	03–185		KP340810	PS65–039	170	71°06.47`S 011°32.29`W
	01–61	KP340845	KP340807	Rothera South Cove	0–30m	65°34’09’S 068°07’54W
***Adacnarca***						
*A*. *nitens A*	01–054		KP340819	Rothera South Cove	0–30m	65°34’09’S 068°07’54W
	03–843	KP340838	KP340834	PS65–326	611	72°51.43`S 019°38.67`W
	01–40–1	KP340842	KP340817	Rothera South Cove	0–30m	65°34’09’S 068°07’54W
	03–560	KP340850	KP340818	PS65–274	289	70°52.16`S 010°43.69`W
*A*. *nitens B*	03–125	KP340847		PS65–039	170	71°06.63`S 011°32.72`W
	03–557		KP340820	PS65–265	290	70°52.74`S 010°52.72`W
	03–645–4		KP340822	PS65–279	120	71°07.48`S 011°29.91`W
	03–815	KP340837	KP340821	PS65–325	457	72°54.76`S 019°43.48`W
*A*. *nitens C*	02–693	KP340851	KP340823	PS61–223	376	60°08.16`S 034°55.59`W
	02–924–1,2	KP340836	KP340825	PS61–217	519	59°54.98`S 032°28.33`W
	02–924–3	KP340836	KP340826	PS61–217	519	59°54.98`S 032°28.33`W
	02–809	KP340843	KP340824	PS61–217	519	59°54.98`S 032°28.33`W
*A*. *nitens D*	02–904		KP340827	PS61–252	287	60°23.45`S 055°16.82`W
*A*. *nitens E*	03-415-3	KP340848	KP340828	PS65–233	846	71°18.99`S 013°56.56`W
*A*. *nitens ‘radials’ F*	03-415-1	KP340848	KP340830	PS65–233	846	71°18.99`S 013°56.56`W
	02–329	KP340839	KP340829	PS61-133-4	1117	65°19.47`S 051°32.55`W
*A*. *nitens ‘radials’ G*	03–767	KP340841	KP340831	PS65–297	650	72°48.50`S 019°31.60`W
	03–798	KP340840	KP340832	PS65–324	670	72°54.52`S 019°47.74`W
*A*. *limopsoides*	03–633	KP340849	KP340833	PS65–278	119	71°07.51`S 011°29.94`W
***Lissarca***						
*L*. *miliaris*	02–834	KP340860	KP340834	Signy Island	0.2	60°43’S 045°36’W
*L*. *notorcadensis*	33		EF192520	WS-787-12	619	72°50’S 019°36’W
	30		EF192519	WS-260-9	241	70°56’S 010°30’W
	31		EF192521	WS-522-11	302	71°05’S 011°33’W
	26		EF192515	WS-198-5	281	70°56’S 010°32’W
	22		EF192509	SO-820–3	401	60°59’S 043°27’W
	6		EF192526	SR-607–6	317	53°24’S 042°42’W
	11		EF192514	SSI-758-2	299	57°41’S 026°26’W
	7		EF192529	SR-729-4	317	53°24’S 042°42’W
	8		EF192530	SR-729-5	317	53°24’S 042°42’W
	10		EF192528	SR-606-3	288	53°23’S 042°41’W
	15		EF192512	SSI-214-4	337	59°43’S 027°57’W
***Cosa***						
*C*. *waikikia*		AB101614				

Specimens were identified to species by shell morphology (*e*.*g*. shape, morphometrics, shell and periostractum patterns, hinge and hinge teeth structure) and subsequently their prodissoconch structure was analyzed to discriminate between Operational Taxonomic Units (OTU) within a species group. OTUs within a species group are designated as ‘A’ to ‘G’ in Table 1. Specimen morphology was studied with a Zeiss Semi SV6 dissecting microscope and a TM3000 scanning electron microscope (SEM).

### Ethics statement

Collections were not made from any protected or private sites within Antarctica. This study did not involve endangered or protected species. All necessary permits were obtained for the described field collections, within the Antarctic Act (1994).

### DNA sequencing

Genomic DNA was extracted from tissue samples using the Qiagen DNeasy Tissue Extraction Kit as directed by the manufacturer. DNA amplification was carried out using the polymerase chain reaction (PCR) with standard reagents. Primer sequences for partial fragments of *18S* (domain 2, LSU 3 and 5) and *28S* rDNA are described in Littlewood [15] and Steiner and Hammer [16]. PCR cycling was carried out in a Thermocycler, with optimized annealing temperatures ranging between 50–55°C. Purification of PCR products was achieved using Qiaquick PCR purification. Approximately 200 ng of double stranded PCR product was used in cycle sequencing reactions following the protocol outlined in the DYEnamic ET Dye Terminator Cycle Sequencing kit for MegaBACE DNA (Amersham Biosciences, Little Chalfont, Buckinghamshire, United Kingdom). Reaction products were visualised on a MegaBACE 500 automated DNA sequencer (Amersham Pharmacia, Little Chalfont, Buckinghamshire, United Kingdom).

All sequences were edited and checked in CodonCode Aligner Version 3.5.6 (CodonCode Corporation 2006). Sequence quality was evaluated using “Phred” quality scores, excluding sequences with values <300 [17,18]. Electropherograms were manually examined for sequencing errors and, where possible, variable positions were confirmed by reference to the corresponding reverse sequences. Fragments of 28S and *18S* were each aligned with arcoid taxa available from earlier studies (‘Arcoida’ datasets, S1 Table) [16]. Pteriomorph outgroups were selected from within Limoidea, Anomioidea and Pterioidea. A combined *18S* and 28S dataset (‘Limopsoidea’ dataset) was also constructed for philobryids only, with *Tegillarca nodifera* and *granosa* (Arcoidea, Arcidae) and pteriod *Pinctada margaritifera* included as outgroups.

### DNA alignment

Alignment was conducted using the program PRANK v100701 [19] with the ‘+F’ option. This is a ‘phylogeny aware’ approach with respect to the accurate placement of insertions and deletions (indels), which is designed to ensure that insertion events are not down-weighted during alignment. This approach is therefore particularly good for aligning sequences with multiple indels [20]. Minor manual adjustments were made by eye following this procedure. ALISCORE v2.0 [21] was then used to determine sections of alignment ambiguity, using Monte Carlo resampling within a sliding window to measure the phylogenetic signal-to-noise ratio compared to a random sample of equivalent size. The program RNAalifold (http://rna.tbi.univie.ac.at/cgi-bin/RNAalifold.cgi) [22] with RIBOSUM scoring was used to estimate a consensus secondary structure from this alignment for subsequent phylogenetic analysis. The combined *18S* and *28S* dataset (Limopsoidea dataset) was constructed by concatenation of the two PRANK alignments for each gene. Base compositional heterogeneity was assessed by χ testing in PAUP 4.0b10.

### Phylogenetic analysis

Maximum likelihood analyses of all datasets were carried out using RaXML (with regions of alignment ambiguity removed if indicated by ALISCORE). Secondary structure models 6A-E, 7A-D and 16A-D were applied, which parameterize rate matrices of varying complexity for paired sites [23]. The 16-state models include evolutionary rate parameters for every possible substitution change between paired bases (*i*.*e*. 4 x 4), while the 6-state models ignore mismatched pairs, so are less parameterized. The secondary structure consensus from RNAalifold was used to determine the nucleotide sites (*e*.*g*. stem, loop) to which the secondary structure models apply. The GTRGAMMA model was employed for loop regions. Node support was measured using 1,000 ‘fast’ bootstrap replicates of the data. Models were compared using Akaike Information Criterion (AIC) scores, derived from the likelihood scores and free parameters counted for each model.

Tests of monophyly of the philobryid genera *Adacnarca* and *Philobrya* and the family Arcidae were carried out using Shimodaira-Hasegawa (SH) testing in PAUP [24]. Maximum likelihood (ML) trees were generated for each dataset using the best fitting model supported by variable-site-corrected Akaike Information Criterion scores in jModelTest v2.1.6 [25]. Each topology was constrained to be monophyletic and then compared with the unconstrained (ML) tree for each locus, using the re-sampling estimated log-likelihood (RELL) method to generate a test distribution [26].

Bayesian analyses of all datasets were conducted in MrBayes v.3.1.2 [27] using the doublet model, which parameterizes the evolutionary rate between doublet pairs in pre-identified stem regions [28]. The locations of each doublet pair were as determined using RNalifold. A simple 4 by 4 rate model was used for loop regions, and gapped sites were ignored. Four Metropolis-Coupled MCMC chains (one cold and three heated) were run simultaneously for 5–10 million generations, with trees sampled at 1000-generation intervals and two replicate runs conducted. Analysis of convergence was assessed by monitoring effective sample size (ESS) estimates for each parameter, using the program TRACER v1.6 [29]. Standard deviation of split frequencies was monitored; analyses were conducted until all values were <0.01, indicating full convergence of runs. The first 25% of runs were discarded as “burn-in”. A 50% majority rule consensus tree was generated from all remaining sampled trees.

### Divergence time analyses

Divergence times were measured using Bayesian relaxed clock analyses in BEAST v1.8.1 [30], using the *28S* Arcoida dataset. Fossil constraints were selected from within the Arcoida.

(1) Glycymeridae: These are suggested to occur from the middle Jurassic (Callovian period) [31], so a minimum divergence time of 161.2 Ma was imposed on the radiation/stem branch for this family, with an exponential distribution of mean size 30 Myr.

(2) Limopsidae: The earliest known limopsid fossil (*Limopsis albiensis*) is from the Early Cretaceous (Albian) [32,33]. We explored the impact of imposing a minimum divergence time of 99.6 Ma on the stem branch for this clade (the upper boundary of the Albian period), using an exponential distribution with mean size 30 Myr.

In order to provide an informative constraint on root height (*i*.*e*. the divergence of arcoids from other pteriomorph bivalves) we examined available fossil data from Pteriomorpha. Earliest pterioid fossils are known from the Ordovician, which suggests the divergence of this outgroup from the ingroup may have been during the Cambrian explosion.

(3) The root height of the tree was therefore constrained to a normal distribution centred in the upper Cambrian (488.3 Ma, standard deviation = 10 Myr), with hard upper and lower boundaries at 542 Ma (*i*.*e*. no older than basal Cambrian) and 455.8 Ma (upper Ordovician) respectively.

An exponential prior distribution was chosen for each in-group constraint with a mean size of 30 Myr, corresponding to upper 95% values of 251.1 Myr and 189.5 Myr respectively for the in-group fossils above. When applying multiple calibrations in divergence time analysis, interaction between the imposed calibration density, underlying tree prior (and associated hyper-parameters), and topological constraints can mean that the marginal prior density on the calibration node is very different from the calibration density imposed [34]. Initial analyses were conducted with priors only (without data, for 50 million generations), in order to determine the prior density distribution on each of the constrained nodes, using a calibrated Yule process [34].

In order to determine the best fitting molecular clock model for this clade, we ran three clock models (strict, exponential relaxed, lognormal relaxed). Analyses were conducted for 30–150 million generations, using a general time reversible plus discrete gamma variation in rates across sites (GTR + G) evolutionary model. In all analyses a calibrated Yule process was used as the tree prior [34]. Following Baele *et al*. [35,36], we selected the best fitting clock model using path and stepping stone sampling (100 path steps over a chain length of 1 million) to calculate marginal likelihood estimates (MLEs), as implemented in BEAST v1.8.1.

Two additional divergence time analyses were conducted.

(4) An early fossil constraint was imposed on the origin time of the Philobryidae, since a assemblage of fossils from the middle Triassic (Anisian period, 237–245 Ma) have been tentatively identified as philobryids due to similar hinge features [37]. If these are early philobryids, this extends the philobryid fossil record much further back than all other fossil evidence, which only goes back to the Eocene [6]. Due to the disjunct nature of this discovery (about 200 million years earlier than other fossil records for this family), we conducted this analysis as a sensitivity to the base case.

(5) For the clock model selected by path sampling, we repeated divergence time analysis removing ingroup fossils, in order to gauge the sensitivity of divergence time results to the fossils applied.

### Species delimitation within Antarctic Philobryidae

In order to delimit species clusters within Philobryidae, we applied two approaches. Firstly, the general mixed Yule coalescent (GMYC) multiple-threshold model [38–40] was conducted using ‘Species Limits by Threshold Statistics’ (SPLITS v1.0–19) in program R (R Project for Statistical Computing: www.r-project.org), as implemented in Monaghan *et al*. [39]. This is a likelihood-based method, which delimits species by fitting within- and between- species branching models to a reconstructed gene tree. The ultra-metric input tree was obtained from the *28S* divergence time analysis described above, applying the molecular clock model most strongly supported according to MLEs. Since nuclear ribosomal genes generally evolve more slowly than their mitochondrial counterparts, this gene is likely to be conservative with respect to species delimitation (*i*.*e*. clusters may be delimited at a higher taxonomic level than they would be if for example the standard barcoding gene *CO1* was used). So single ‘species’ clusters measured by *28S* may be resolved as multiple species clusters using the same GMYC method with mitochondrial DNA. This analysis therefore provides a conservative (minimum) measure of the number of likely philobryid species in the dataset. Secondly, a test for intraspecific divergence based on the ‘barcoding gap’ was also applied to this dataset [41], using the ABGD web server (http://wwwabi.snv.jussieu.fr/public/abgd/abgdweb.html). Prior maximum divergence of intraspecific diversity *P* was investigated over a range of 0.001–0.015. The maximum number of groups identified across this range is reported, since the *28S* dataset is likely to delimit groups at a higher taxonomic level than for example *CO1*. So where species are placed into multiple distinct groups, then this provides strong evidence that they are distinct species, but species that cluster together cannot be conclusively considered a single species by delimitation with these markers.

## Results

**Fig 2:**
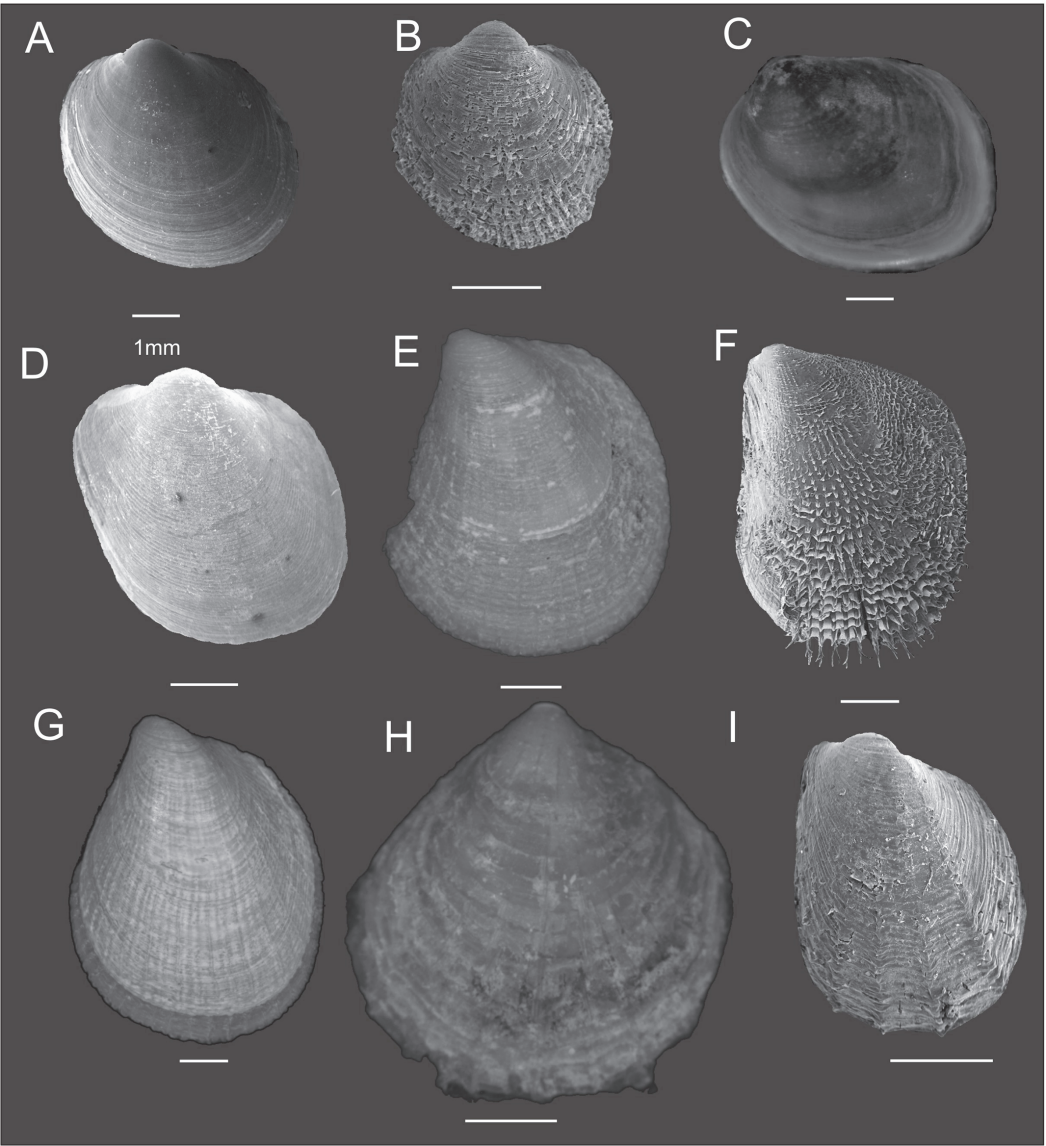
Philobryid shell morphology. Specimens labeled A-I refer to (A) *Adacnarca nitens*, (B) *A*. *limopsoides* (C) *Lissarca miliaris* (D) *L*. *notorcadensis* (E) *Philobrya capillata* (F) *P*. *crispa* (G) *P*. *magellanica*, (H) *P*. *sublaevis* and (I) *P*. *wandelensis*.

**Fig 3:**
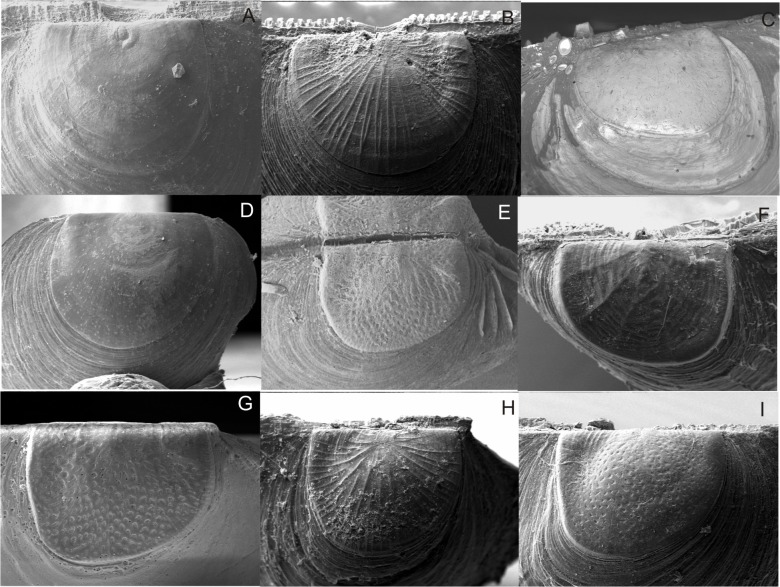
Philobryid prodissoconch morphology. For species identifications refer to Fig. 2.

The 34 philobryid specimens sequenced in this study belong to three genera (*Adacnarca*, *Lissarca*, *Philobrya*) and were assigned to 18 species and three species-groups (*A*. *nitens*, *P*. *magellanica* and *P*. *wandelensis*) based on their morphological characters (Figs. 2 and 3, S2 Table). Morphological analysis results are described in the (S1 Text).

### Molecular sequence data

Aligned partial *18S* and *28S* sequences of lengths 1221bp and 1175bp were obtained from a broad range of depths across the South Atlantic (Fig. 1). The *18S* dataset was composed of two fragments (998bp and 217bp respectively), since some sequences failed to amplify in the intervening region. Assessment of alignment ambiguity with ALISCORE revealed that all regions had sufficient levels of phylogenetic signal relative to noise, so no sections were excluded. Basic statistics for the three alignments are shown in Table 2, and indicate that *28S* is more variable than *18S* within the Arcoida (249 and 93 parsimony informative sites respectively). Base compositional heterogeneity was not detected in any datasets.

**Table 2:** Philobryid *18S* and *28S* evolutionary genetic parameters and tests of family and genus level monophyly.

Taxonomic level	Arcoidea	Arcoidea	Philobryidae
Locus	*18S*	*28S*	*18S+28S*
Alignment length (bp)	1221	1175	2294
Taxa	51	69	32
Variable sites	192	400	355
Parsimony informative sites	93	249	204
**Monophyly tests**			
Evolutionary model	GTR+I+G	GTR+I+G	TIM1+I+G
LnL of ML tree	3539.36	6414.19	5926.49
Difference from ML LnL			
*Adacnarca*	0.00	0.00	0.00
*Philobrya*	0.22	2.77	5.21
*Arcoida*	N/A	0.13	N/A

Note: No monophyly tests were significant. ML refers to Maximum Likelihood and LnL refers to log likelihood. Evolutionary model abbreviations: GTR = general time reversible, TIM1 = “Transitional” model with unequal base frequencies, G = gamma, I = invariant sites, for description see [42].

### Phylogenetic relationships

**Fig 4:**
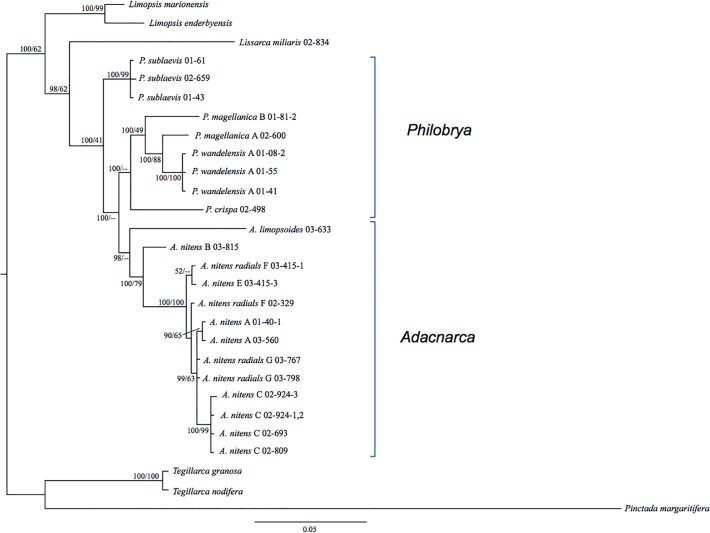
Molecular phylogeny of *18S+28S*. Node values show Bayesian posterior probabilities (as %) and maximum likelihood bootstrap support respectively. All alignments and phylogenetic trees associated with these figures can be downloaded from TreeBase (http://treebase.org, submission 16834).

The Limopsoidea (*18S*+*28S*) dataset (Fig. 4) was used to determine the evolutionary relationships within the Philobryidae since it contained the most variable sites. AIC comparisons of the RAXML analyses (S3 Table) revealed secondary structure model 6A to be best fitting to this dataset. Bayesian doublet analysis provided much stronger posterior support for key nodes than maximum likelihood, providing >0.95 posterior support for all inter-species nodes. Three key clades are identified in the dataset considering both morphological species designations and genetic clusters: the *Adacnarca nitens* complex plus *A*. *limopsoides* (0.98 Bayesian posterior probability, BPP), a sister clade uniting *P*. *wandelensis*, *magellanica* and *crispa* (1.00 BPP), and a basal clade uniting *P*. *sublaevis* (1.00 BPP).

Small numbers of variable sites ‘diagnostic’ for each of these clades were counted using the *18S* and *28S* arcoid datasets, with one identified for the *Adacnarca* complex (in *28S*), one for the *P*. *wandelensis*/*magellanica/crispa* cluster (*28S*), and 4 for *sublaevis* (*28S*).

#### *Adacnarca* clade

**Fig 5:**
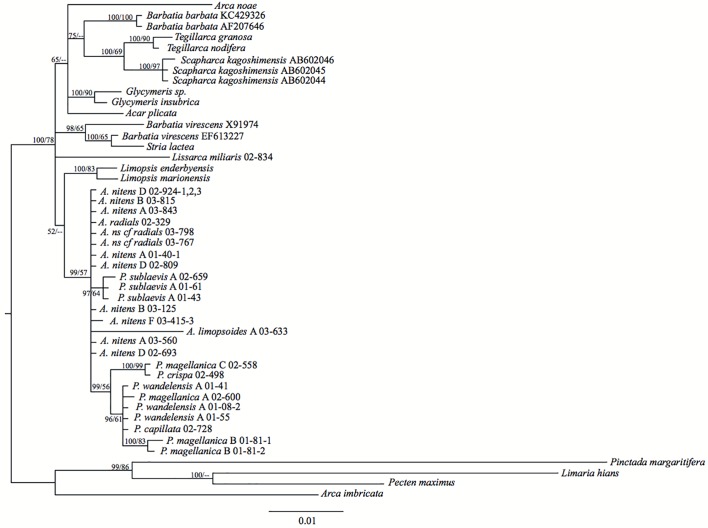
Molecular phylogeny of *18S*. Node values show Bayesian posterior probabilities (as %) and maximum likelihood bootstrap support respectively.

**Fig 6:**
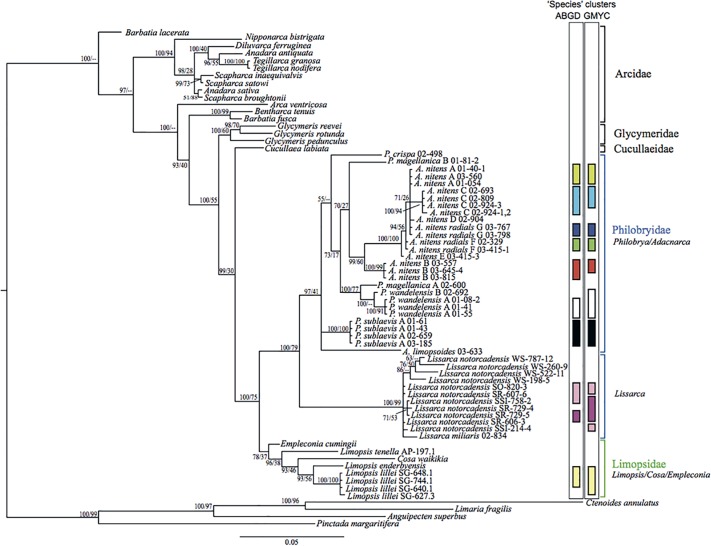
Molecular phylogeny of *28S*. Node values show Bayesian posterior probabilities (as %) and maximum likelihood bootstrap support respectively. Coloured bars show species units identified using ABGD and GMYC.

The monophyly of this genus is strongly supported by the *18S*+*28S* dataset only (0.98 BPP) and by SH testing of both gene datasets (Table 2). Within the *18S*+*28S* phylogeny, *A*. *limopsoides* is placed basal to *A*. *nitens* with >0.95 BPP. Within the *18S* phylogeny, *Adacnarca* and *A*. *nitens* form an unresolved polytomy with other Philobrya taxa (Fig. 5). Within the *28S* phylogeny, *A*. *nitens* is a strongly supported monophyletic group (0.99 BPP, Fig. 6), but the placement of *A*. *limopsoides* is more basal within the Philobryidae, suggesting a polyphyletic *Adacnarca*. Divergence time analysis reveals rate variation across *Adacnarca*, particularly on the *A*. *limopsoides* branch, which has an elevated mutation rate relative to other philobryids. The long branch subtending this taxon likely explains the instability of this taxon across the *28S* phylogenetic and divergence time analyses.

#### *Philobrya wandelensis/magellanica/crispa* clade

The monophyly of this clade is strongly supported by the *18S*+*28S* and *18S* datasets (1.00 and 0.99 BPP respectively) (Figs. 4–5). The *28S* phylogeny places *P*. *crispa* and *P*. *magellanica* B polyphyletic with *Adacnarca* (Fig. 6). However, as above, divergence time analysis of *28S* provides relationships more concordant with the *18S*+*28S* hypothesis, grouping this clade as a monophyletic unit. As with *A*. *limopsoides*, *P*. *crispa* has a slightly elevated evolutionary rate relative to other *Philobrya*, so rate variation may have influenced the basal placement of this taxon in the *28S* MrBayes tree. Overall the placement of *P*. *crisp*a basal to other *Philobrya* in this clade seems the most likely hypothesis. In *18S*+*28S* and *28S* analyses, *P*. *wandelensis* A is a strongly resolved monophyletic group, with *P*. *wandelensis* B placed as a sister taxon. *P*. *magellanica* A and B are resolved as paraphyletic lineages basal to this clade with 1.00 BPP. *P*. *magellanica* C and *P*. *capillata* were only characterized for *18S*, and the poor within-family resolution of this locus means no inference on the evolutionary affinities of these taxa can be made at present.

#### *Philobrya sublaevis* clade

This clade is strongly supported (>0.95 BPP) in all analyses. It is placed basal to the *Adacnarca* and other *Philobrya wandelensis/magellanica/crispa* clades in the combined *18S*+*28S* analysis, as well as the *28S* divergence time analyses (Figs. 4 and 6). SH testing of the overall monophyly of *Philobrya* (*P*. *sublaevis* plus *P*. *wandelensis/magellanica/crispa*) did not reject a monophyletic hypothesis. However this hypothesis is rejected by Bayesian analysis of the *18S*+*28S* dataset, which strongly supports a basal position for the *sublaevis* clade.

#### Arcoid interrelationships

There are insufficient taxa to address this with the combined *18S*+*28S* analysis. The philobryid genus *Lissarca* is placed as a strongly supported sister group to the *Philobrya/Adacnarca* clade in the *28S* analysis, but is not strongly supported by *18S* alone, where only one *Lissarca* specimen is included. The *28S* Arcoida dataset strongly supports a monophyletic Philobryidae consisting of *Philobrya*, *Adacnarca* and *Lissarca* (1.00 BPP). The sister group relationship of Limopsidae + Philobryidae relative to other members of Arcoida is strongly supported by the *28S* dataset (1.00 BPP), supporting the taxonomic classification of these families into superfamily Limopsoidea [6]. Phylogenetic relationships differ between the divergence time scenarios, with Limopsidae placed closer to Arcidae when a Triassic divergence date is imposed on the Philobryidae, and placed as sister taxon to the Philobryidae when this divergence date is not imposed. However strong *28S* posterior support for the latter set of relationships suggests this is the more likely hypothesis.

Similarly, the relationship between Cucullaeidae and Glycymeridae is also influenced by fossil timings. For the *28S* molecular phylogenetic analysis, the sister groups of Limopsoidea are resolved as Cucullaeidae and then Glycymeridae with strong support (1.00 BPP). The grouping of Glycymeridae with the Limopsoidea has been hypothesized previously [6] but questioned by Malchus and Warén [43] based on hinge and ligament development. This specific phylogenetic grouping of Glycymeridae and Cucullaeidae as separate sister groups to the Limopsoidea has not been proposed previously. Interestingly, when a Triassic origin for the Philobryidae is imposed, the Glycymeridae plus Cucullaeidae are placed as a sister taxon to the Arcidae instead, with 0.99 BPP support for this grouping.

**Fig 7:**
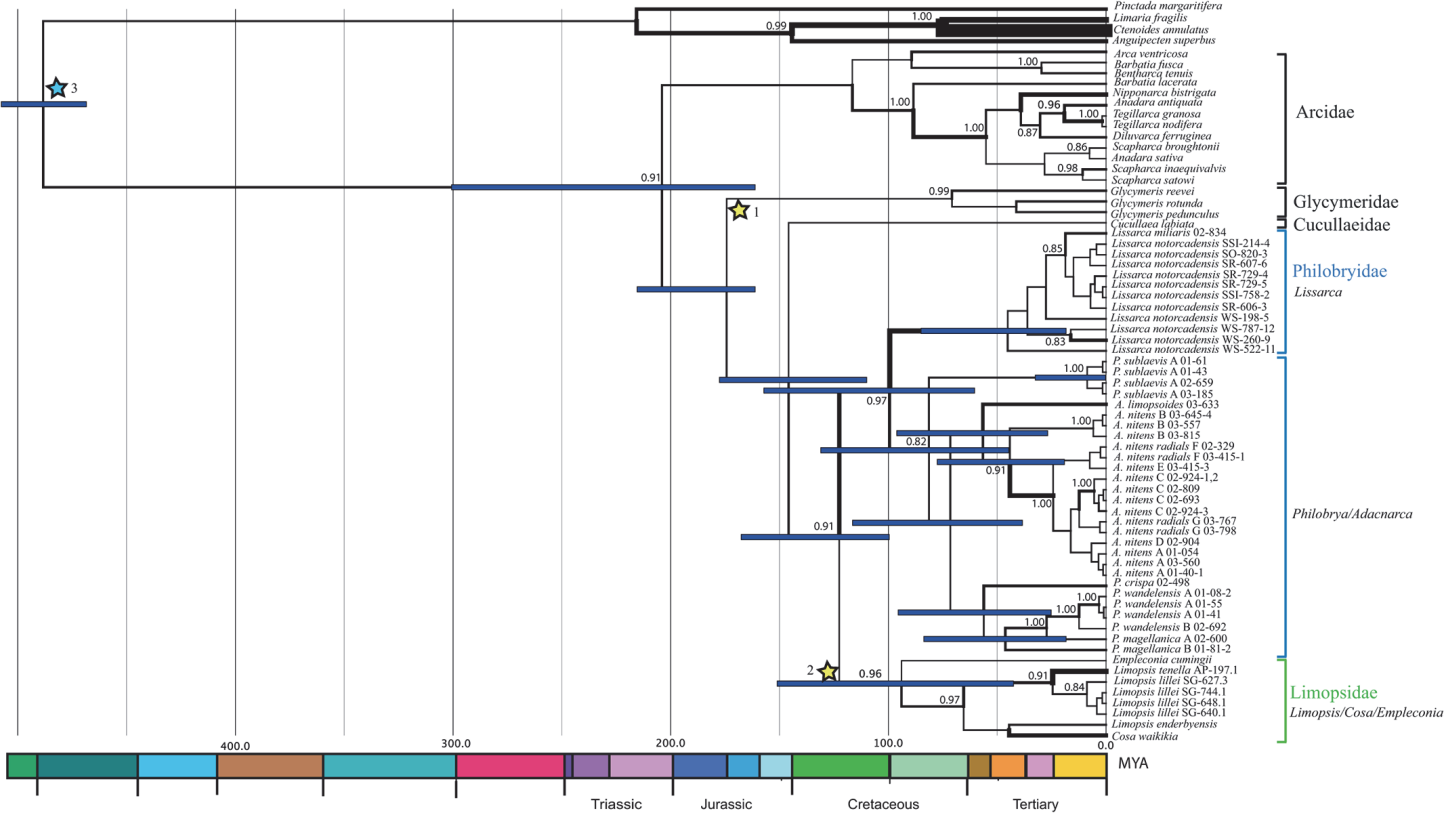
Divergence time analysis using *28S* with multiple fossil constraints and an uncorrelated lognormal relaxed clock. Rate variation across the phylogeny is depicted using branch thickness. Posterior support values over 80% are shown. Bars at key nodes represent 95%-iles on estimated divergence times. Fossil constraints are indicated by stars with details given in the text.

While the Bayesian analysis of *28S* produced a polyphyletic Arcidae (Fig. 6), SH testing did not reject the monophyly of Arcidae for any of the datasets analysed (Table 2), and this family is reconstructed as a monophyletic group with divergence time analysis (Fig. 7). There is some evidence supporting the existence of superfamily Arcoidea (here comprising Arcidae, Cucullaeidae and Glycymeridae, with no sampling of Noetiidae) [44]. SH testing does not reject this hypothesis, but at present taxonomic sampling is too limited to make further inferences about arcoid phylogenetic groupings.

### Divergence times

Path sampling yielded very similar log marginal likelihood scores for the uncorrelated lognormal and exponential relaxed clocks (scores −6875.7 and −6876.7 respectively). The lognormal clock was therefore very marginally favoured, with a likelihood difference of 0.91. Both were much better fitting than the strict clock model (-6939.5). Stepping stone sampling also yielded very similar scores for the uncorrelated lognormal and exponential relaxed clocks, with an even smaller difference between the two models (−6876.3 and −6876.2 respectively) slightly favouring the exponential clock. Given the slightly larger likelihood difference between models obtained with path sampling, we chose to conduct GMYC and additional divergence time analyses with the lognormal clock, noting however that the difference in support between the clock models is negligible.

Divergence time analysis with only the root height constrained and no additional ingroups yielded very recent divergence estimates among all ingroup taxa, spanning 50 Ma. Overall, there appears to be a rate slowdown within the arcoid ingroup relative to other pteriomorph outgroups, as shown by this result and the higher rates attributed to the outgroups when ingroup fossils are imposed (Fig. 1 and S1 Fig.). Further sampling of additional pteriomorph taxa will be required to investigate this rate variation in more detail.

When ingroup fossils were used (excepting the Triassic philobryids), both exponential and lognormal clock models place the radiations of all Southern Ocean philobryid species within the Paleogene (Fig. 7). Within *Adacnarca nitens*, morpho-species B is the first to diverge at 45 Ma, concurrently also the date of first radiation of the *P*. *magellanica/ wandelensis* and *L*. *notorcadensis/ miliaris* clades. Higher taxonomic level splits within *Adacnarca* and *Philobrya* occur following the K-Pg boundary, around 60 Ma for both *A*. *limopsoides/ nitens* and *P*. *crispa/ magellanica/ wandelensis*. The *Limopsis* group diverged more recently, with *tenella/ lillei* species diverging around 30 Ma. The *P*. *sublaevis* clade divergences begin around 5 Ma. This analysis dates the origin time of Philobryidae to the early Cretaceous, a great deal subsequent to the Triassic occurrence documented by Stiller and Jinhua [37]. If the Philobryidae have Triassic origins, the common time of origination of *A*. *nitens*, the *P*. *magellanica/wandelensis* clade, *Limopsis* and *Lissarca notorcadensis/miliaris* would be the K-Pg boundary (66 Ma) (S1 Fig.). Interestingly, the times of divergence within Arcidae were similar regardless of the additional constraint, while divergences between *Philobrya* and *Adacnarca* genera occurred earlier, during the early to mid-Cretaceous. Radiations within shelf-associated *A*. *nitens*, *P*. *sublaevis* and *P*. *wandelensis* still occur throughout the Miocene.

### Species limits

Since species delimitation is applied to *28S* rather than the *CO1* barcoding gene, the classifications are used here as a guide to groupings likely to contain one or more unique species. Species limits identified by ABGD and GMYC analysis were mostly concordant, both with each other and with the morphological identifications. ABGD analysis recovered *A*. *nitens* morpho-species A to G. The GMYC analysis split *A*. *nitens* B and C each into two units. Morphological identifications of *Philobrya sublaevis* and *magellanica* were concordant with both analytical approaches. ABGD was consistent with morphological analysis in splitting *wandelensis* into two units, but GMYC grouped all *wandelensis* taxa as a single cluster. Likelihood ratio testing within the GMYC model for a shift between Yule branching (species) and coalescent (population) processes was significant (LnL difference of 6.8), indicating that GMYC clustering results reflect a significant shift in the pattern of branching within the phylogeny.

## Discussion

Here we present the first molecular study of the Philobryidae, a poorly known family which is also one of the most speciose bivalve families in the Southern Ocean. Our investigation into the evolutionary history and morphology of this family represents an important first step towards identifying the environmental characteristics that have enabled these species to diversify and thrive in high latitude Antarctic waters. Although divergence time estimates differ depending on whether *Eophilobryoidella sinoanisica* is considered a fossil philobryid [37], both scenarios indicate that the widely distributed Southern Ocean philobryid *A*. *nitens* diverged prior to the point when the Southern Ocean began to cool and Antarctic ice sheets were formed [45]. It should be noted that these dates are also sampling dependent, so it is always possible that earlier radiation dates are resolved with further sampling of these taxa. However divergence dates cannot become more recent with additional sampling, only older. The Triassic philobryid divergence scenario suggests that radiation of *A*. *nitens* began at the K-Pg boundary (66 Ma) while the alternate scenario places the *A*. *limopsoides/ A*. *nitens* split at the K-Pg boundary, with the *A*. *nitens* radiation following in the middle Eocene. Since both species are endemic, this may be an exclusively Southern Ocean radiation. However the relationships between the taxa in this study and other unsampled philobryid species are unknown, so Southern Ocean specificity may not be exclusive throughout this period. Further taxon sampling will be required to resolve this question.

The radiations of the two other exclusively Antarctic species, *wandelensis* and *sublaevis* are estimated to be much more recent, during the mid Miocene (10–15 Ma), following the establishment of the Antarctic circumpolar current and development of Antarctic ice sheets. These estimates are similar over both divergence time scenarios and are consistent with recent evidence that a remnant volcanic arc in the Scotia Sea may have formed a barrier to eastward dispersal prior to the mid-Miocene [46].

The divergence of the *wandelensis* from the Magellanic species *magellanica* is estimated in the mid-Oligocene around the time that the Drake Passage opened, possibly reflecting the introgression of this species into the Southern Ocean current around that time. A similar time frame was estimated for octopus genus *Paraledone*, which was estimated to diverge into the Southern Ocean from the deep sea in the Oligocene [47]. In this case however *P*. *magellanica* species (Magellanic to sub-Antarctic) were found at similar depths to polar *P*. *wandelensis*, discounting the hypothesis of species emergence onto the Southern Ocean shelf from deeper waters for this genus. Our divergence time estimates for these brooding bivalves are consistent with the ‘ACC’ hypothesis put forward by Pearse *et al*. [48] that strong currents through the Drake Passage over the past 30 My have dislodged and transported Magellanic brooding species into new locations in the Scotia Sea and beyond. This hypothesis implies that the diversity of brooders should decline with distance from the Scotia Sea. This cannot be evaluated with the current dataset but more widespread Southern Ocean and Magellanic sampling of philobryids will enable this question to be addressed. Recently, Poulin *et al*. [49] measured divergence time estimates between South America and the Southern Ocean for a selection of brooding and planktotrophic species, finding that many diverged close to the Miocene-Pliocene boundary. While more recent than the estimates presented here, these estimates are consistent with the Drake Passage ACC as a transporting mechanism.

Philobryids form a monophyletic group, with the three genera (*Philobrya*, *Adacnarca* and *Lissarca*) falling into four distinct clades, and *Philobrya* split between a *sublaevis* clade and a *crispa/ magellanica/ wandelensis* clade. While a monophyletic origin for these two clades was not rejected by SH testing, Bayesian analysis provided strong posterior support for these as polyphyletic within the Philobryidae, suggesting that a taxonomic revision of this genus into two genera may be required. Given the limited taxon sampling of this family, we assume the patterns revealed in this study reflect at least four incursions and radiations of philobryid species into Southern Ocean waters (*P*. *wandelensis*, *A*. *nitens*, *P*. *sublaevis* and *L*. *notorcadensis*). More exhaustive taxon sampling is required to derive a full biogeography of the Philobryidae and determine whether these multiple radiations derive from a common ancestor in the Southern Ocean or elsewhere.

Oliver and Holmes observed that families classified into Arcoida are supported by very few synapomorphic characters, and noted the general problem of widespread homoplasy within the Order [44]. Our analysis corroborates previous morphological classifications, by placing the Philobryidae as the sister group to Limopsidae within the Arcoida. The relationships between these families and Glycymeridae and Cucullaeidae are not clearly resolved with this phylogeny; *28S* groups Limopsoidea as a sister taxon to *Cucullaea*, and then to Glycymeridae, both with >0.95 BPP support. Nicol [50] proposed that Glycymeridae evolved from cucullaeids, but this molecular phylogeny suggests the order of origination may have been the opposite way round. Malchus and Warén suggested that the Glycymerididae originated from a duplivincular taxon which might belong to the Cucullaeidae [43]. Further arcoid gene sequencing and taxon sampling will be required to characterize these relationships with more certainty.

Nearly all of the philobryids examined in this study were collected from the continental shelf (100–700m depth), with a couple of the most recently evolving clades collected exclusively from very shallow waters (*P*. *sublaevis* A 01–61 and 01–43, *A*. *nitens* morpho-species A). Only one philobryid clade is associated with deeper water; morpho-species *A*. *nitens radials* E and F were collected between 800–1150m. The ordinal relationships among the *A*. *nitens* morphospecies in the divergence time analysis suggest the possibility of deep-water emergence of *A*. *nitens* onto the Southern Ocean shelf from the middle Oligocene onwards. In this analysis we do not sample from the full geographic range of *A*. *nitens*, so additional collections will help to understand the origin and diversification of this clade in more detail.

Our findings of greater cryptic diversity than previously supposed for Philobryidae are consistent with many other studies of benthic brooders in the Southern Ocean [51] and further illuminate the hidden biodiversity of the Southern Ocean benthos [2]. While philobryids such as *Adacnarca nitens* [9] are able to crawl short distances, longer distance dispersal has been proposed via rafting with other organisms, possibly facilitated by ice scouring of their biotic substrates [52]. The opening of the Drake Passage around the Eocene-Oligocene transition (∼34 Ma) is likely to have facilitated the further divergences seen within *A*. *nitens* and *wandelensis*. Further population level study of these clades and sequencing of additional rapidly evolving mitochondrial DNA markers is expected to throw more light on the key drivers underscoring within-species divergence.

## Supporting Information

S1 Text Morphological identifications of Antarctic Philobryidae (DOCX)

S1 Table Additional pteriomorph taxa included in *18S* and *28S* analyses (DOCX)

S2 Table Morphological characteristics of philobryid specimens (DOCX)

S3 Table AIC_c_ support for secondary structure models in RAXML (DOCX)

S1 Fig Divergence time analysis with multiple fossil constraints, including Triassic philobryid. Rate variation across the phylogeny is depicted using branch thickness. Posterior support values over 80% are shown. Bars at key nodes represent 95%-iles on estimated divergence times. Fossil constraints are starred, see text for details.
(EPS)
