# Supplementary material for: The Evolutionary Origins of the Southern Ocean Philobryid Bivalves: Hidden Biodiversity, Ancient Persistence
Source: PLoS One. 2015 Apr 8;10(4):e0121198. doi: 10.1371/journal.pone.0121198 (PMC4390230; doi:10.1371/journal.pone.0121198)
Supplement: S1 Text — (DOCX) [file pone.0121198.s001.docx]

**S1 Text. Morphological identifications of Antarctic Philobryidae**

*Adacnarca nitens* group

The type locality of *Adarcnarca nitens* (Pelseneer, 1903) [[1](#_ENREF_1)] is in the east Antarctic Davis Sea. Since its original description, it was recorded by numerous studies from the Antarctic continental shelf and from islands in the Southern Ocean [[2 and references therein](#_ENREF_2)]. The species is small (< 8mm), circular to obliquely rounded, white-ish with a smooth surface and holds few outer morphological characters. Egorova [[3](#_ENREF_3)] described *Adacnarca polarsterni* from the shelf of the eastern Weddell Sea living sympatric with *A. nitens* based on differences in shell morphology, *e.g.* an oval shell shape (height<length) and anterior and posterior second dentition of almost equal length. The specimens analysed in this study showed second dentition characteristics of the posterior tooth plate longer than the anterior one and were assigned to *A. nitens*.

The SEM analysis of prodissoconch (PD) characters (length, height, ratio length/height) separated the studied specimens into 8 morphological groupings: A) PDl ~420-450µm, PDh ~327-383 µm, Rl/h 1.19-1.29, B) PDl ~208-240µm, PDh ~143-168 µm, Rl/h 1.43-1.45, C) PDl ~630-690µm, PDh ~540-580 µm, Rl/h 1.14-1.26, D) PDl ~418µm, PDh ~390 µm, Rl/h 1.07; E) PDl ~503µm, PDh ~428 µm, Rl/h 1.17, F) PDl ~537-572µm, PDh ~500-511 µm, Rl/h 1.07-1.11, faint radial pattern on dissoconch, G) PDl ~371-372µm, PDh ~321-328 µm, Rl/h 1.13-1.15, faint radial pattern on dissoconch.

*Adacnarca limopsoides* (Thiele, 1912)

The type locality of *Adarcnarca limopsoides* [[4](#_ENREF_4)] is the Gauss Station in the east Antarctic. The species has a circum-Antarctic distribution [[5](#_ENREF_5)]. The species is small (< 5mm), rounded with prominent prodissoconch, shell covered with thick brown periostracum with broad hairs. The SEM analysis of prodissoconch (PD) characters (length, height, ratio length/height) found one grouping with PDl ~571-639µm, PDh ~420-533 µm, Rl/h 1.20-1.38.

*Lissarca miliaris* (Philippi, 1845)

The type locality of *Lissarca miliaris* [[6](#_ENREF_6)] is the Strait of Magellan in the South American Magellan region. The species has been recorded from the Magellan region, Falkland Islands, South Georgia, Kerguelen, South Orkney and South Shetland Islands and northern Antarctic Peninsula [[7](#_ENREF_7)] but not from the high Antarctic continental shelf. The species is small (< 5mm), trapezoidal and covered with a reddish-brown periostracum. The variability in shell morphology, prodissconch size and reproduction between populations of *L. miliaris* has recently been published [[7-9](#_ENREF_7)]. Investigating populations from Borge Bay on Signy Island collected over 36 years, Reed *et al*. [[8](#_ENREF_8)] reported a trend of increasing shell growth rate and a decrease in prodissoconch size while the annual average temperatures in the area increased.

*Lissarca notorcadensis* group

*Lissarca notorcadensis* (Melvill and Standen, 1907 [[10](#_ENREF_10)]) is widely distributed in the Southern Ocean, including, circum-Antarctic on the continental shelf, on the Antarctic islands, and also reported from the Kerguelen and Magellan Region [[2](#_ENREF_2)]. The species is small (< 10 mm), trapezoidal to subquadrate and covered with a white periostracum. Variability in shell morphometrics and the COI gene were reported by Cope and Linse [[11](#_ENREF_11)] and Linse *et al.* [[12](#_ENREF_12)] for specimens from the Scotia Arc, Ross and Weddell seas.

*Philobrya* *capillata* (Dell 1964)

The specimen assigned to *Philobrya* *capillata* was collected off Shag Rocks. It resembles in shell and prodissoconch morphology *Philobrya capillata*, which was described from the Shag Rocks, the Falkland Islands and Palmer Archipelago [[13](#_ENREF_13)]. The species is large (>8 mm), pinctatoid and has a thin brown periostractum with multiple radial lines with short hair-like bristles. Engl [[5](#_ENREF_5)] speculates that the specimen from the Palmer Archipelago might be *P. tumida*.

*Philobrya crispa* (Linse 2002)

The species is known from the Magellan Region and Falkland Islands, and its type locality is off Isla Picton in the Beagle Channel [[14](#_ENREF_14)]. The species is small (< 5 mm), pinctadoid and has a distinct brown periostracum with long, curved hair-like bristles. The shell morphology (shell morphometric, periostracum structure, prodissoconch) of this specimen collected from the Falkland Islands matches *P. crispa*.

*Philobrya magellanica* – group

The type locality of *Philobrya magellanica* (Stempell, 1899) is Punta Arenas in the Strait of Magellan [[15](#_ENREF_15)] and the species is reported from the Magellan Region and the Falkland Islands [[16](#_ENREF_16)]. The species is relatively large (> 8 mm), subpyriform and the hyaline periostracum overlaps the shell edge. While the dissoconch and periostracum morphology is similar to *P. sublaevis*, the prodissoconch patterns are distinctly different between the two species: *P. magellanica* has a pitted pattern while *P. sublaevis* has > 10 radial ribs.

The SEM analysis of prodissoconch (PD) characters (length, height, ratio length/height) separated the studied specimens into 3 morphological groupings: A) PDl ~565µm, PDh ~486 µm, Rl/h 1.16, B) PDl ~368-436µm, PDh ~275-343 µm, Rl/h 1.26-1.34 and C) PDl ~836µm, PDh ~626 µm, Rl/h 1.34.

*Philobrya sublaevis* (Pelseneer, 1903)

The *Philobrya sublaevis* type locality is in the east Antarctic Davis Sea [[1](#_ENREF_1)] and since then the species has been collected from around the Antarctic continent and islands in the Southern Ocean [[5](#_ENREF_5),[16](#_ENREF_16)]. The species has several accepted synonymies reported in Dell [[16](#_ENREF_16)] and Aldea and Troncoso [[2](#_ENREF_2)]. *P. sublaevis* is relatively large (>8 mm), subpyriform, the hyaline periostracum overlaps the shell edge and it has a haired, radial pattern.

The studied specimens were consistent in dissoconch and prodissoconch morphology described for *P. sublaevis*. The SEM analysis of prodissoconch (PD) characters (length, height, ratio length/height) found one grouping with PDl ~629-693µm, PDh ~528-584 µm, Rl/h 1.19-1.25.

*Philobrya wandelensis* –group

The type locality of *Philobrya wandelensis* (Lamy, 1906) is Wandel Island on the Antarctic Peninsula. This species is widely distributed around the Antarctic continent and on the islands in the Southern Ocean [[2](#_ENREF_2)]. The species is small (< 5 mm), pinctadoid and has a brown periostracum, which has 5-7 radials with short, broad hairs.

The SEM analysis of prodissoconch (PD) characters (length, height, ratio length/height) separated the studied specimens into 2 morphological groupings: A) PDl ~557-639µm, PDh ~449-533 µm, Rl/h 1.20-1.38 and B) PDl ~418 µm; height not measurable because of erosion.

1. Pelseneer P (1903) Results du voyage du S. Y. "Belgica" 1897-1899. Rapports Scientifiques, Zoologie, Mollusques (Amphineures, Gastropodes et Lamellibranches). Anvers.

2. Aldea C, Troncoso JS (2008) Systematics and distribution of shelled molluscs (Gastropoda, Bivalvia and Scaphopoda) from the South Shetland Islands to the Bellingshausen Sea, West Antarctica. Iberus 26: 1-75.

3. Egorova EN (2003) New species of the genus *Adacnarca* (Bivalvia, Philobryidae) from Antarctica. Zoologicheskii Zhurnal 82: 771-780.

4. Thiele J (1912) Die antarktischen Schnecken und Muscheln. In: Drygalski E, editor. Deutsche Südpolar-Expedition (1901-1903), Berlin, Zoologischer Band 5, Heft 2 pp. 183-286.

5. Engl W (2012) Shells of Antarctica: Hackenheim: Conchbooks. 402 p.

6. Philippi RA (1845) Abbildungen und Beschreibungen neuer oder wenig bekannter Conchylien. Archiv für Naturgeschichte, Berlin 3: 33-64.

7. Reed AJ, Linse K, Thatje S (2014) Differential adaptations between cold-stenothermal environments in the bivalve *Lissarca cf. miliaris* (Philobryidae) from the Scotia Sea islands and Antarctic Peninsula. J Sea Res 88: 11-20.

8. Reed A, Thatje S, Linse K (2012) Shifting baselines in Antarctic ecosystems; Ecophysiological response to warming in *Lissarca miliaris* at Signy Island, Antarctica. PLoS One 7: e53477.

9. Reed AJ, Thatje S, Linse K (2013) An unusual hermaphrodite reproductive trait in the Antarctic brooding bivalve *Lissarca miliaris* (Philobryidae) from the Scotia Sea, Southern Ocean. Polar Biol 36: 1-11.

10. Melvill JC, Standen R (1907) The marine Mollusca of the Scottish National Antarctic Expedition. Trans Roy Soc Edinburgh 46: 119-157.

11. Cope T, Linse K (2006) Morphological differences in *Lissarca notorcadensis* Melvill and Standen, 1907 from the Scotia, Weddell and Ross Seas. Deep-Sea Res Pt Ii 53: 903-911.

12. Linse K, Cope T, Lorz AN, Sands C (2007) Is the Scotia Sea a centre of Antarctic marine diversification? Some evidence of cryptic speciation in the circum-Antarctic bivalve *Lissarca notorcadensis* (Arcoidea : Philobryidae). Polar Biol 30: 1059-1068.

13. Dell RK (1964) Antarctic and sub-Antarctic Mollusca Amphineura, Scaphopoda and Bivalvia. Discovery Reports 33.

14. Linse K (2002) The shelled Magellanic Mollusca: with special reference to biogeographic relations in the Southern Ocean. Ruggell, Liechtenstein. 251 p.

15. Stempell W (1899) Die Muscheln der Sammplung Plate. Fauna Chilensis Zoologische Jahrbücher 4: 217-250.

16. Dell RK (1990) Antarctic Mollusca with special reference to the fauna of the Ross Sea. Bulletin of the Royal Society of New Zealand 27: 1-311.
