# Supplementary material for: The Evolutionary Origins of the Southern Ocean Philobryid Bivalves: Hidden Biodiversity, Ancient Persistence
Source: PLoS One. 2015 Apr 8;10(4):e0121198. doi: 10.1371/journal.pone.0121198 (PMC4390230; doi:10.1371/journal.pone.0121198)
Supplement: S1 Table — (DOCX) [file pone.0121198.s002.docx]

**S2 Table. Additional pteriomorph taxa included in 18S and 28S analyses**

| Taxon | *18S* | *28S* |
| --- | --- | --- |
| Outgroups | *Pinctada margaritifera:* AJ389638 | *Pinctada margaritifera:* AB214466 |
|  | *Pecten maximus*: PCTRRE | *Anguipecten superbus*: AB102746 |
|  | *Limaria hians*: AF120534 | *Limaria fragilis*: AB102742 |
|  |  | *Ctenoides annulatus*: AJ307550 |
| Arcoida | *Tegillarca granosa*: EF613230 | *Tegillarca granosa*: AB101602 |
|  | *Tegillarca nodifera*: EF613229 | *Tegillarca nodifera*: AB101603 |
|  | *Stria lactea*: AF120531 |  |
|  | *Arca imbricata*: AY654986 | *Arca ventricosa*: AB101612 |
|  | *Arca noae*: X90960 |  |
|  | *Acar plicata*: AJ389630 |  |
|  | *Barbatia virescens*: X91974 | *Barbatia fusca*: AB101606 |
|  | *Barbatia virescens*: EF613227 | *Barbatia lacerata*: AB101605 |
|  | *Barbatia barbata*: KC429326 |  |
|  | *Barbatia barbata*: AF207646 |  |
|  |  | *Bentharca tenuis*: AB101607 |
|  | *Scapharca kagoshimensis*: AB602046 | *Scapharca inaequivalvis*: AB101596 |
|  | *Scapharca kagoshimensis*: AB602045 | *Scapharca satowi*: AB101597 |
|  | *Scapharca kagoshimensis*: AB602044 | *Scapharca broughtonii*: AB101599 |
|  |  | *Anadara sativa*: AB101598 |
|  |  | *Anadara antiquata:* AB101601 |
|  |  | *Nipponarca bistrigata*: AB101604 |
|  |  | *Diluvarca ferruginea*: AB101600 |
| Glycymeridae | *Glycymeris sp*.: X91978 | *Glcymeris reevei*: AB101609 |
|  | *Glycymeris insubrica*: AF207647 | *Glycymeris pedunculus*: AJ307534 |
|  |  | *Glycymeris rotunda:* AB101608 |
| Cucullaeidae |  | *Cucullaea labiata*: AB101611 |
| Limopsidae | *Limopsis enderbyensis:* AJ422057 | *Limopsis enderbyensis:* AY321301 |
|  | *Limopsis marionensis:* AJ422058 |  |
|  |  | *Limopsis lillei*: AY319801 (648-1) |
|  |  | *Limopsis lillei*: AY319807 (744-1) |
|  |  | *Limopsis lillei*: AY319808 (640-1) |
|  |  | *Limopsis lillei*: AY319804 (627-3) |
|  |  | *Limopsis tenella*: AY321302 (197) |
|  |  | *Empleconia cumingii:* AB101610 |
|  |  | *Cosa waikikia*: AB101614 |
