# Supplementary material for: The Evolutionary Origins of the Southern Ocean Philobryid Bivalves: Hidden Biodiversity, Ancient Persistence
Source: PLoS One. 2015 Apr 8;10(4):e0121198. doi: 10.1371/journal.pone.0121198 (PMC4390230; doi:10.1371/journal.pone.0121198)
Supplement: S2 Table — (DOCX) [file pone.0121198.s003.docx]

**S3 Table. Morphological characteristics of philobryid specimens**

|  | *A. nitens* A | *A. nitens* B | *A. nitens* C | *A. nitens* D | *A. nitens* E | *A. nitens* F radials | *A. nitens* G radials | *A. limopsoides* | *L. miliaris* | *L. notorcadensis* | *P. capillata* | *P. crispa* | *P. magellanica* A | *P. magellanica* B | *P. magellanica* C | *P. sublaevis* | *P. wandelensis* A | *P. wandelensis* B |
| --- | --- | --- | --- | --- | --- | --- | --- | --- | --- | --- | --- | --- | --- | --- | --- | --- | --- | --- |
| **Shell outline** |  |  |  |  |  |  |  |  |  |  |  |  |  |  |  |  |  |  |
| circular to obliquely rounded | x | x | x | x | x | x | x | x |  |  |  |  |  |  |  |  |  |  |
| trapezoidal to subquadrate |  |  |  |  |  |  |  |  | x | x |  |  |  |  |  |  |  |  |
| pinctadoid |  |  |  |  |  |  |  |  |  |  | x | x |  |  |  |  | x | x |
| subpyriform |  |  |  |  |  |  |  |  |  |  |  |  | x | x | x | x |  |  |
| **Shell size** |  |  |  |  |  |  |  |  |  |  |  |  |  |  |  |  |  |  |
| > 8 mm |  |  |  |  |  |  |  |  |  |  | x |  | x | x | x | x |  |  |
| **Periostracum** |  |  |  |  |  |  |  |  |  |  |  |  |  |  |  |  |  |  |
| overhanging shell |  |  |  |  |  |  |  |  |  |  |  |  | x | x | x | x |  |  |
| smooth | x | x | x | x | x | x | x |  | x | x |  |  |  |  |  |  |  |  |
| interspaced radial hairy ridges |  |  |  |  |  |  |  |  |  |  | x |  | x | x | x | x | x | x |
| dense hairy ridges |  |  |  |  |  |  |  | x |  |  |  | x |  |  |  |  |  |  |
| short hairs |  |  |  |  |  |  |  | x |  |  |  |  |  |  |  |  | x | x |
| long hairs |  |  |  |  |  |  |  |  |  |  |  | x |  |  |  | x |  |  |
| **Prodissoconch** |  |  |  |  |  |  |  |  |  |  |  |  |  |  |  |  |  |  |
| smooth | x | x | x | x | x | x | x |  | x | x |  |  |  |  |  |  |  |  |
| 5-7 radials |  |  |  |  |  |  |  |  |  |  |  | x |  |  |  |  |  |  |
| >10 radials |  |  |  |  |  |  |  | x |  |  |  |  |  |  |  | x | x | x |
| pitted |  |  |  |  |  |  |  |  |  |  | x |  | x | x | x |  |  |  |
| commarginals |  |  |  |  |  |  |  |  |  |  |  | x |  |  |  |  |  |  |
| **Hinge** |  |  |  |  |  |  |  |  |  |  |  |  |  |  |  |  |  |  |
| symmetric |  |  |  |  |  |  |  | x |  | x |  |  |  |  |  |  |  |  |
| asymmetric, anterior shorter | x | x | x | x | x | x | x |  | x |  | x | x | x | x | x | x | x | x |
| ligament groove rectangular |  |  |  |  |  |  |  | x |  |  |  |  |  |  |  |  |  |  |
| lig. groove symmetric triangular |  |  |  |  |  |  |  |  | x | x |  |  |  |  |  |  |  |  |
| lig. groove asymmetric triangular | x | x | x | x | x | x | x |  |  |  | x | x | x | x | x | x | x | x |
| second dentition posterior teeth plate longer | x | x | x | x | x | x | x |  | x |  | x | x | x | x | x | x | x | x |
| second dentition ant. & post. teeth subequal | x | x | x | x | x | x | x |  | x | x |  |  |  |  |  |  |  |  |
| sec. dent. ant. centrally longer, post. subequal |  |  |  |  |  |  |  |  |  |  | x | x | x | x | x | x | x | x |
| second dentition ant. & post. centrally longer |  |  |  |  |  |  |  | x |  |  |  |  |  |  |  |  |  |  |
| third dentition present |  |  |  |  |  |  |  | x | x | x |  | x |  |  |  | x | x | x |
