# Supplementary material for: The Evolutionary Origins of the Southern Ocean Philobryid Bivalves: Hidden Biodiversity, Ancient Persistence
Source: PLoS One. 2015 Apr 8;10(4):e0121198. doi: 10.1371/journal.pone.0121198 (PMC4390230; doi:10.1371/journal.pone.0121198)
Supplement: S3 Table — (DOCX) [file pone.0121198.s004.docx]

**S4 Table. AIC_c_ support for secondary structure models in RAXML by dataset**

| Model | Stem parameters | Loop parameters | 28S | | | 18S | | | 18S+28S | | |
| --- | --- | --- | --- | --- | --- | --- | --- | --- | --- | --- | --- |
|  |  |  | LnL | Total free parameters | AIC | LnL | Total free parameters | AIC | LnL | Total free parameters | AIC |
| 6A | 20 | 9 | -4913 | 164 | **10154** | -2479 | 118 | 5194 | -4359 | 86 | **8882** |
| 6B | 8 | 9 | -4963 | 152 | 10230 | -2490 | 106 | **5193** | -4382 | 74 | 8904 |
| 6C | 5 | 9 | -4965 | 149 | 10228 | -2498 | 103 | 5202 | -4382 | 71 | 8898 |
| 6D | 3 | 9 | -4981 | 147 | 10257 | -2498 | 101 | 5198 | -4397 | 69 | 8924 |
| 6E | 7 | 9 | -4979 | 151 | 10260 | -2498 | 105 | 5206 | -4397 | 73 | 8932 |
| 7A | 27 | 9 | -5877 | 171 | 12097 | -3025 | 125 | 6299 | -4896 | 93 | 9970 |
| 7B | 24 | 9 | -5876 | 168 | 12088 | -3025 | 122 | 6293 | -4896 | 90 | 9964 |
| 7C | 16 | 9 | -5883 | 160 | 12085 | -3031 | 114 | 6290 | -4907 | 82 | 9971 |
| 7D | 10 | 9 | -5944 | 154 | 12195 | -3067 | 108 | 6351 | -4970 | 76 | 10083 |
| 7E | 8 | 10 | -5952 | 153 | 12210 | -3071 | 107 | 6356 | -4990 | 75 | 10123 |
| 7F | 7 | 11 | -5947 | 153 | 12200 | -3068 | 107 | 6349 | -4970 | 75 | 10081 |
| 16A | 20 | 9 | -6382 | 164 | 13091 | -3211 | 118 | 6657 | -5166 | 86 | 10497 |
| 16B | 17 | 9 | -6809 | 161 | 13940 | -3478 | 115 | 7187 | -5423 | 83 | 11004 |

AIC_c_ is calculated as (-2LnL+(2*total free parameters). ‘Total free parameters’ sums all secondary structure model parameters plus total branch length parameters for each dataset (135 for 28S, 89 for 18S, 57 for 18+28S).
